# A Predictive Scoring Model for Postoperative Tracheostomy in Patients Who Underwent Cardiac Surgery

**DOI:** 10.3389/fcvm.2021.799605

**Published:** 2022-01-28

**Authors:** Dashuai Wang, Su Wang, Yifan Du, Yu Song, Sheng Le, Hongfei Wang, Anchen Zhang, Xiaofan Huang, Long Wu, Xinling Du

**Affiliations:** ^1^Department of Cardiovascular Surgery, Union Hospital, Tongji Medical College, Huazhong University of Science and Technology, Wuhan, China; ^2^Department of Emergency Medicine, Union Hospital, Tongji Medical College, Huazhong University of Science and Technology, Wuhan, China; ^3^Department of Cardiology, The Central Hospital of Wuhan, Tongji Medical College, Huazhong University of Science and Technology, Wuhan, China

**Keywords:** tracheostomy, cardiac surgery, risk factors, prediction model, risk score

## Abstract

**Background:**

A subset of patients require a tracheostomy as respiratory support in a severe state after cardiac surgery. There are limited data to assess the predictors for requiring postoperative tracheostomy (POT) in cardiac surgical patients.

**Methods:**

The records of adult patients who underwent cardiac surgery from 2016 to 2019 at our institution were reviewed. Univariable analysis was used to assess the possible risk factors for POT. Then multivariable logistic regression analysis was performed to identify independent predictors. A predictive scoring model was established with predictor assigned scores derived from each regression coefficient divided by the smallest one. The area under the receiver operating characteristic curve and the Hosmer-Lemeshow goodness-of-fit test were used to evaluate the discrimination and calibration of the risk score, respectively.

**Results:**

A total of 5,323 cardiac surgical patients were included, with 128 (2.4%) patients treated with tracheostomy after cardiac surgery. Patients with POT had a higher frequency of readmission to the intensive care unit (ICU), longer stay, and higher mortality (*p* < 0.001). Mixed valve surgery and coronary artery bypass grafting (CABG), aortic surgery, renal insufficiency, diabetes mellitus, chronic obstructive pulmonary disease (COPD), pulmonary edema, age >60 years, and emergent surgery were independent predictors. A 9-point risk score was generated based on the multivariable model, showing good discrimination [the concordance index (c-index): 0.837] and was well-calibrated.

**Conclusions:**

We established and verified a predictive scoring model for POT in patients who underwent cardiac surgery. The scoring model was conducive to risk stratification and may provide meaningful information for clinical decision-making.

## Introduction

Respiratory pulmonary complications are common after cardiac surgery, such as pneumonia and acute respiratory failure, which are associated with an increase in morbidity, mortality, and healthcare utilization (1–3). Postoperative respiratory failure has been previously reported with an incidence of 9.1–15.9% in patients who underwent cardiac surgery (3, 4), and patients with severe respiratory failure after cardiac surgery require tracheostomies. Tracheostomy is generally regarded as a marker of serious postoperative outcomes, and two-thirds of the deaths occur in patients who underwent tracheostomy after cardiac surgery (5).

Despite most critically ill patients with respiratory failure tolerating short-term tracheal intubation well with few complications, longer mechanical ventilation is related to adverse outcomes, and tracheostomy as a common critical care procedure is used for these patients who require prolonged mechanical ventilatory support (6). Renal failure, aortic procedures, and hemodynamic instability have been revealed to predict the occurrence of respiratory failure after cardiac surgery (4). Moreover, risk factors for prolonged mechanical ventilation in patients who underwent cardiac surgery have been identified, such as age more than 65 years, emergency surgery, and left ventricular ejection fraction of 30% or less (7). However, not all the patients who need prolonged mechanical ventilation are tracheostomized. Predictor assessment of tracheostomy among cardiac surgical patients in the early stage is limited, and a predictive model for tracheostomy is still in urgent need.

In this study, we mainly aimed to identify independent predictors for postoperative tracheostomy (POT) in patients who underwent cardiac surgery and to develop and validate a predictive scoring model. The model may be useful for risk identification and decision-making in perioperative management.

## Methods

### Ethical Statement

Ethical approval was given by the Medical Ethics Committee of Tongji Medical College, Huazhong University of Science and Technology (IORG No. IORG0003571). The study protocol was in accordance with the Declaration of Helsinki. Patient informed consent was waived.

### Study Population

This was a retrospective study performed in a tertiary medical center that included adult patients who underwent cardiac surgery between January 2016 and December 2019. We involved all cardiac surgical patients with the exception of those who had immunosuppression, immunodeficiency, organ transplantation, intraoperative death, discharge or death within 48 h after surgery, and incomplete medical records.

### Baseline Characteristics

All clinical data of this study population were extracted from the electronic medical-record system of our institution. The demographic variables that were assessed in this study were gender, age, height, weight, body mass index, smoking history, and drinking history. Preoperative comorbidity variables that were evaluated in the study were hypertension, diabetes mellitus, pulmonary edema, cerebrovascular disease, chronic obstructive pulmonary disease (COPD), past history of cardiac surgery, past history of general history, renal insufficiency, gastrointestinal tract disease, peripheral vascular disease, atrial fibrillation, pulmonary artery hypertension, pericardial effusion, left ventricular ejection fraction, the New York Heart Association (NYHA) class III–IV, diameter of the left atrium, diameter of the left ventricle, diameter of the right atrium, and diameter of the right ventricle. Laboratory variables in our analysis contained serum creatinine, serum albumin, serum globulin, platelet count, white blood cell count, red blood cell count, and hemoglobin. The different surgical types that were analyzed included isolated valve surgery, isolated coronary artery bypass grafting (CABG), mixed valve surgery and CABG, aortic surgery, other types, and emergent surgery.

Postoperative variables included readmission to the intensive care unit (ICU), the length of ICU stay and hospital stay, and in-hospital mortality.

### Endpoints

The primary endpoint of this study was tracheostomy after cardiac surgery. All the tracheostomies were performed by the percutaneous route. The indications for tracheostomy in this study included prolonged mechanical ventilation, one or more failed trails of extubation, repeated intubation, predicted difficult reintubation, bypass of upper airway obstruction, and the need for tracheal access to remove thick pulmonary secretions (8). The secondary endpoints were readmission to ICU, in-hospital mortality, and the lengths of ICU and hospital stay.

### Statistical Analysis

Statistical analysis was performed using IBM SPSS (version 24, Armonk, NY, USA). Normally distributed continuous variables were shown as means ± SDs, while non-normally distributed continuous variables as medians and inter-quartile ranges. Categorical variables were expressed as counts with percentages. Data with normal distribution were analyzed by Student's *t*-test, while data with non-normal distribution were analyzed by Mann-Whitney *U*-test. Categorical variables were analyzed by χ^2^ tests or Fisher's exact test.

Univariable analysis was conducted to assess the possible risk factors for POT among all the collected variables. Those factors associated with tracheostomy after cardiac surgery (exploratory *p* < 0.10) were introduced into the multivariable logistic regression analysis, which was used to identify independent risk factors for POT. Then, the significant continuous variables were dichotomized according to the Youden index or the cutoff values reported previously, and multivariable logistic analysis was performed again. In this multivariable model, the independent predictors were assigned with scores, which were rounded up integers, derived from each regression coefficient divided by the smallest one. The scores of all the predictors were summed to generate a total risk score for one person, and risk subgroups of predicting POT were constructed based on the composite risk score.

**Figure 1:**
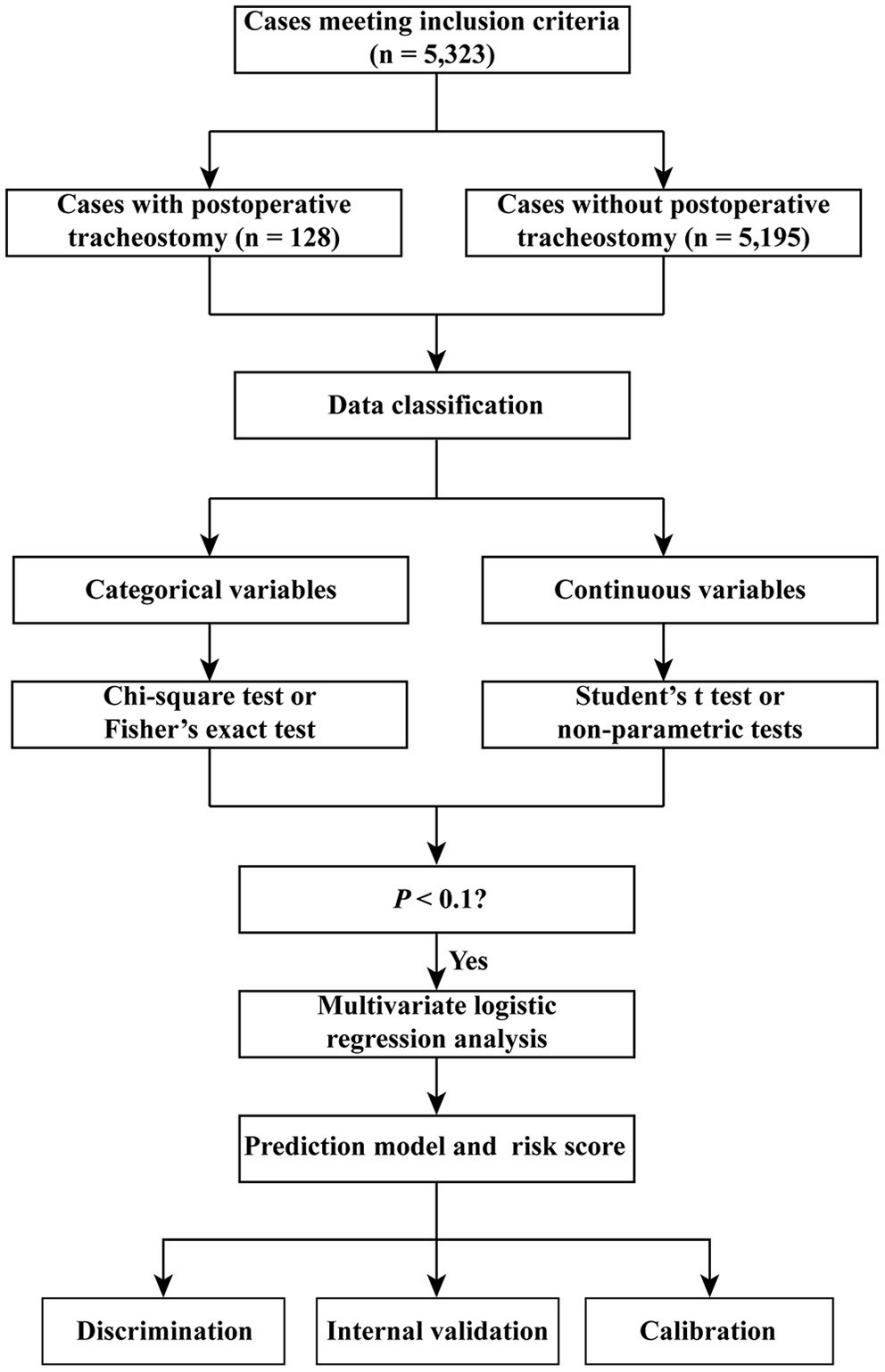
Flow chart of the study.

The area under the receiver operating characteristic (ROC) curve (AUC) or the concordance index (c-index) was used to evaluate the discrimination of the risk score. The AUCs of the three models were compared: the first model with continuous variables, the second model with the dichotomy of the continuous variables, and the third model with risk scores. The Hosmer-Lemeshow goodness-of-fit test was conducted to assess the calibration of the model. *p* < 0.05 was considered statistically significant. The flow chart of the study is presented in Figure 1.

## Results

### Baseline Characteristics

A total of 5,323 patients who underwent cardiac surgery were included in this study for analysis. The average age was 51 years; 51.4% of the patients were men (Table 1). The average body mass index was 23.4 kg/m^2^; more than 20% of the patients had a drinking or smoking history. The most common underlying conditions were peripheral vascular disease (42.4%), followed by cerebrovascular disease (34.0%), hypertension (30.1%), past history of general history (28.1%), and pulmonary artery hypertension (26.1%). A minority of patients had preoperative pulmonary edema (4.8%) and past history of cardiac surgery (7.1%). The most commonly performed surgical type was isolated valve surgery (54.4%). The proportion of isolated CABG, mixed valve surgery and CABG, and emergent surgery were around 10%. Intraoperative variables are presented in Supplementary Table 1.

**Table 1:** Univariable analysis of possible risk factors for POT after cardiac surgery.

**Characteristics**	**Without POT *n* = 5,195 (%)**	**With POT *n* = 128 (%)**	***P*-value**
Demographics			
Male	2,907 (56.0)	94 (73.4)	<0.001
Age (years)	51.03 ± 12.90	56.95 ± 12.04	<0.001
Height (cm)	164.95 ± 8.03	165.97 ± 8.51	0.084
Weight (kg)	63.76 ± 11.97	67.65 ± 14.64	0.006
Body mass index (kg/m^2^)	23.32 ± 3.37	24.42 ± 4.20	0.008
Smoking history	1,489 (28.7)	59 (46.1)	<0.001
Drinking history	1,117 (21.5)	37 (28.9)	0.045
Underlying conditions			
Hypertension	1,529 (29.4)	75 (58.6)	<0.001
Diabetes mellitus	417 (8.0)	21 (16.4)	0.001
Chronic obstructive pulmonary disease	536 (10.3)	21 (16.4)	0.026
Cerebrovascular disease	1,750 (33.7)	62 (48.4)	0.001
Peripheral vascular disease	2,208 (42.5)	50 (39.1)	0.437
Atrial fibrillation	896 (17.2)	16 (12.5)	0.159
Renal insufficiency	471 (9.1)	53 (41.4)	<0.001
Gastrointestinal tract disease	434 (8.4)	13 (10.2)	0.468
Pulmonary edema	242 (4.7)	11 (8.6)	0.039
Past history of cardiac surgery	363 (7.0)	13 (10.2)	0.167
Past history of general history	1,464 (28.2)	33 (25.8)	0.551
NYHA class III–IV	852 (16.4)	24 (18.8)	0.479
Pulmonary artery hypertension	1,370 (26.4)	20 (15.6)	0.006
Pericardial effusion	732 (14.1)	24 (18.8)	0.136
Diameter of the left atrium (cm)	4.2 (3.6, 5.0)	4.0 (3.6, 4.8)	0.222
Diameter of the left ventricle (cm)	5.0 (4.5, 5.7)	4.9 (4.5, 5.6)	0.359
Diameter of the right atrium (cm)	3.8 (3.5, 4.4)	3.8 (3.5, 4.4)	0.940
Diameter of the right ventricle (cm)	3.6 (3.3, 4.0)	3.6 (3.4, 4.1)	0.316
Left ventricular ejection fraction (%)	62 (58, 66)	62 (59, 65)	0.236
Laboratory values			
White blood cell count (×10^9^/L)	5.78 (4.78, 7.10)	7.48 (5.47, 11.11)	<0.001
Red blood cell count (×10^12^/L)	4.28 (3.92, 4.64)	4.18 (3.74, 4.65)	0.076
Hemoglobin (g/l)	129 (118, 140)	127 (114, 141)	0.340
Platelet count (×10^9^/L)	180 (145, 221)	156 (120, 197)	<0.001
Serum albumin (g/L)	40.5 (38.0, 42.7)	39.0 (36.2, 41.4)	<0.001
Serum globulin (g/L)	24.4 (21.7, 27.2)	25.3 (21.9, 28.5)	0.081
Serum creatinine (μmol/L)	71.7 (60.8, 85.0)	82.3 (69.4, 114.0)	<0.001
Surgical types			<0.001
Isolated valve surgery	2,871 (55.3)	26 (20.3)	
Isolated coronary artery bypass grafting	576 (11.1)	15 (11.7)	
Mixed valve surgery and coronary artery bypass grafting	463 (8.9)	18 (14.1)	
Aortic surgery	840 (16.2)	64 (50.0)	
Other types	445 (8.5)	5 (3.9)	
Emergent surgery	437 (8.4)	55 (43.0)	<0.001

*POT, postoperative tracheostomy*.

### Derivation of the Risk Score Model

**Figure 2:**
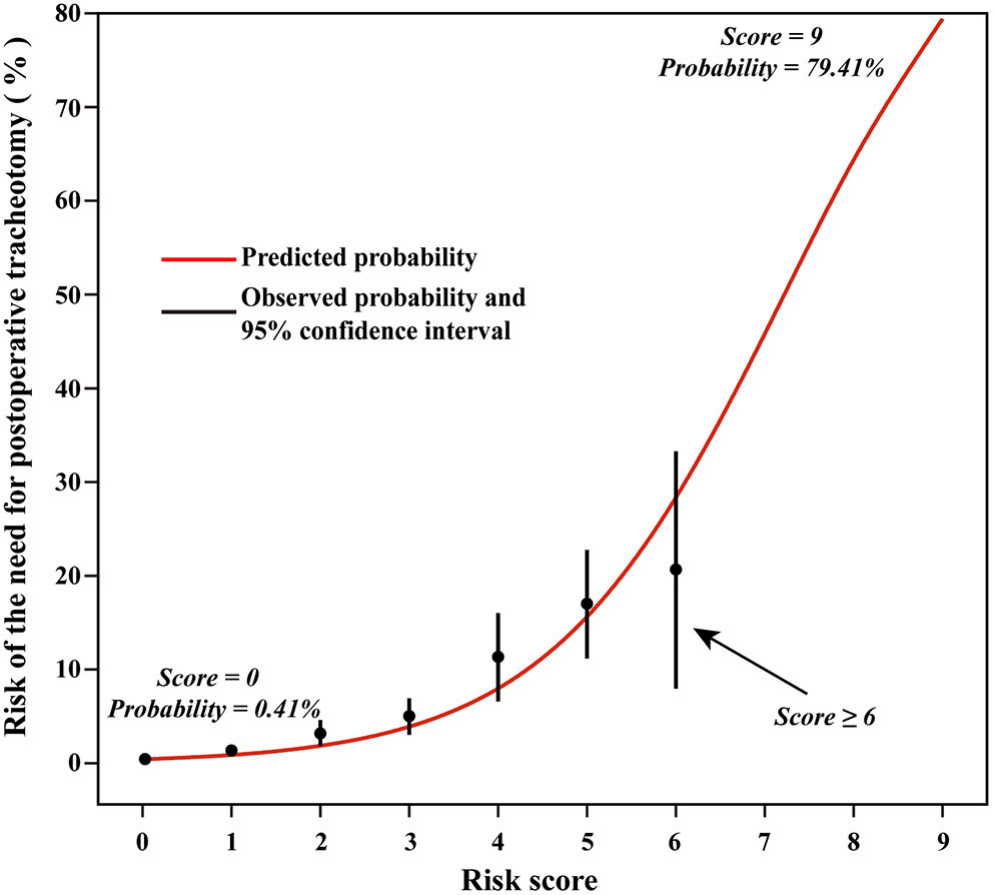
Predicted rates of the need for postoperative tracheostomy based on the risk score model.

There were 128 (2.4%) patients treated with tracheostomy after cardiac surgery. The possible risk factors in univariable analysis were entered into multivariable regression analysis, such as surgical types, renal insufficiency, diabetes mellitus, COPD, pulmonary edema, age, and emergent surgery (Table 2). Significant factors in the first multivariable model were mixed valve and CABG surgery, aortic surgery, renal insufficiency, diabetes mellitus, COPD, pulmonary edema, older age, and emergent surgery. After dichotomizing the variables, we obtained the second multivariable model, among which the age >60 years was an independent predictor (Table 3). Predictors were assigned 1 or 2 points according to their regression coefficients in the second model, and thus, the third model with risk score was derived (Table 3). There were overall 9 points of the risk score and the predicted probabilities of POT based on the risk score are presented in Figure 2.

**Table 2:** Multivariable analysis of independent risk factors for POT after cardiac surgery, without dichotomy of the continuous variables.

**Characteristics**	**OR (95% CI)**	***P*-value**	**Coefficient**
Surgical types		0.022	
Isolated valve surgery	Reference	Reference	Reference
Isolated coronary artery bypass grafting	1.857 (0.922–3.743)	0.083	0.619
Mixed valve surgery and coronary artery bypass grafting	2.446 (1.302–4.597)	0.005	0.895
Aortic surgery	2.685 (1.254–5.749)	0.011	0.988
Other types	2.240 (0.839–5.983)	0.108	0.807
Renal insufficiency	3.386 (2.277–5.034)	<0.001	1.220
Diabetes mellitus	2.089 (1.222–3.571)	0.007	0.737
Chronic obstructive pulmonary disease	2.644 (1.554–4.499)	<0.001	0.972
Pulmonary edema	2.853 (1.438–5.660)	0.003	1.048
Age (years)	1.043 (1.024–1.062)	<0.001	0.042
Emergent surgery	5.244 (2.503–10.987)	<0.001	1.657
Constant	0.001	<0.001	−7.483

*CI, confidence interval; OR, odds ratio; POT, postoperative tracheostomy*.

**Table 3:** Multivariable analysis of independent risk factors for POT after cardiac surgery, with dichotomy of the continuous variables.

**Characteristics**	**OR (95% CI)**	***P*-value**	**Coefficient**	**Point value**
Surgical types		0.019		
Isolated valve surgery	Reference	Reference	Reference	0
Isolated coronary artery bypass grafting	1.978 (0.976–4.010)	0.058	0.682	0
Mixed valve surgery and coronary artery bypass grafting	2.673 (1.421–5.026)	0.002	0.983	1
Aortic surgery	2.523 (1.176–5.416)	0.018	0.926	1
Other types	1.859 (0.700–4.936)	0.213	0.620	0
Renal insufficiency	3.352 (2.247–5.000)	<0.001	1.210	2
Diabetes mellitus	2.210 (1.286–3.799)	0.004	0.793	1
Chronic obstructive pulmonary disease	2.740 (1.608–4.669)	<0.001	1.008	1
Pulmonary edema	2.864 (1.443–5.686)	0.003	1.052	1
Emergent surgery	5.315 (2.525–11.186)	<0.001	1.671	2
Age >60 years	2.136 (1.441–3.167)	<0.001	0.759	1
Constant	0.004	<0.001	−5.481	

*CI, confidence interval; OR, odds ratio; POT, postoperative tracheostomy*.

### Validation and Assessment of the Risk Score Model

**Figure 3:**
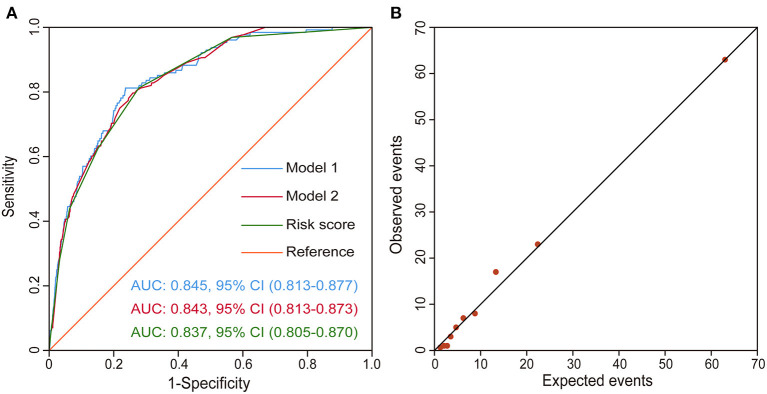
Validation of the risk score model. **(A)** ROC curves drawn using the risk score model, Model 1, and Model 2 in the derivation set. Model 1 is the first model with continuous variables and Model 2 is the second model with a dichotomy of the continuous variables. **(B)** Observed vs. expected events by predicted risk category. The correlation between observed and predicted events is 0.99. CI, confidence interval; ROC curve, receiver operating characteristic curve.

The discriminatory performance of each model was assessed by the AUC. The AUC of the first multivariable model was 0.845 (95% CI, 0.813–0.877), indicating very good discriminatory ability. Similarly, the second multivariable model had an AUC of 0.843 (95% CI, 0.813–0.873). Comparing the predictive abilities of these two models, we found that there was no significant difference (*p* = 0.945), which meant that it was appropriate to dichotomize continuous variables as the dichotomy did not significantly reduce the predictive ability of the model. The AUC of the third model, the risk score model, was 0.837 (95% CI, 0.805–0.870), also showing robust predictive performance. There appeared no significant difference in the predictive ability after comparing those three ROC curves pair by pair (*p* > 0.5) indicated that the predictive ability was not significantly reduced after the latter two model processing, which was reasonable and was more conducive to clinical application. The three ROC curves are presented in Figure 3A. The model showed good calibration by both visual inspection and goodness-of-fit test (Hosmer-Lemeshow χ^2^ = 4.270, *p* = 0832; Figure 3B).

### Risk Stratification

Based on the calculated composite risk score, three risk intervals were identified as low-risk, medium-risk, and high-risk groups. Patients with scores ≤ 3 as a low-risk group constituted 92.8% of the total study population, having a rate of 1.5% to require POT. Patients with scores 4–5 as a medium-risk group constituted 6.4% of the total study population, having a rate of 14.0% to require POT. While, patients with scores ≥6 as a high-risk group constituted 0.8% of the total study population, having a rate of 19.5% to require POT.

### Outcomes

The whole mortality of the study population was 3.1%, and the patients with POT had significantly higher mortality than patients without POT (46.9 vs. 2.1%, *p* < 0.001; Table 4). Similarly, higher frequency of readmission to ICU, longer ICU, and hospital stay were observed in patients with POT.

**Table 4:** Postoperative variables in patients with and without POT after cardiac surgery.

**Variables**	**All patients** ***n* = 5,323 (%)**	**Without POT** ***n* = 5,195 (%)**	**With POT** ***n* = 128 (%)**	***P*-value**
Readmission to ICU	207 (3.9)	163 (3.1)	44 (34.4)	<0.001
ICU stay (days)	3 (2, 5)	3 (2, 5)	22 (18, 31)	<0.001
Hospital stay (days)	15 (11, 19)	14 (11, 19)	34 (24, 47)	<0.001
Mortality	167 (3.1)	107 (2.1)	60 (46.9)	<0.001

*ICU, intensive care unit; POT, postoperative tracheostomy*.

## Discussion

Tracheostomy may improve comfort and reduce the need for sedation and analgesia in patients who require prolonged mechanical ventilation in the ICU compared with translaryngeal airway access and can also provide more favorable conditions for the weaning of mechanical ventilation (9). In our study population who underwent cardiac surgery, the overall incidence of POT was 2.4%, which was within the range of the results in previous studies (10, 11). However, from our results, the cardiac surgical patients treated with POT were more likely to encounter readmission to ICU, experience a longer ICU and hospital stay, and have a higher mortality rate. The mortality of patients who underwent tracheostomy in this study could reach 46.9%, in line with reported in-hospital mortality 36–49% before (5, 12). The survival after the development of prolonged mechanical ventilation is interactively affected by factors, such as age, preoperative pulmonary dysfunction, and chronic renal insufficiency, and the implementation of tracheostomy appears to be a sign for patients less likely to wean from mechanical ventilation (5, 13). In light of these findings, early identification of high-risk patients who require tracheostomy warranted investigations, and a targeted strategy implemented to improve clinical outcomes was needed.

In the current study, we found that preoperative risk factors, such as mixed valve and CABG surgery, aortic surgery, renal insufficiency, diabetes mellitus, COPD, pulmonary edema, older age, and emergent surgery were independently related to POT in patients who underwent cardiac surgery. To our knowledge, this is the first study with a large sample size to identify preoperative predictors for POT after cardiac surgery. A scoring model to predict the probability of POT was further constructed and was validated with good predictive performance, which might early and accurately select the patients who would develop into a severe condition needing POT treatment.

Emergent surgery and pre-existing renal insufficiency were two strong predictors with a higher score in our scoring model. In cardiac surgical patients, emergent surgery was identified as an independent risk factor for the development of ventilatory dependency by Murthy et al. and about 26% of those experiencing ventilatory dependency underwent tracheostomy (13). Beverly et al. also identified that emergency surgery and chronic kidney disease were associated with a high incidence of reintubation after cardiac surgery (14). Patients with chronic kidney disease appeared to have attenuated cardiopulmonary adaptations, which might reflect the affected respiratory muscle strength (15). In addition, kidney disease-related exercise intolerance could lead to increased frailty and worsened quality of life, while the frailty among patients who received mechanical ventilation was related to the need for tracheostomy (16, 17). Moreover, frailty and sarcopenia often co-occur in patients with older age, and sarcopenia can result in impaired ability to take deep breaths and clear secretions (17). Our study showed that older patients who underwent cardiac surgery were at greater risk of developing severe respiratory failure requiring tracheostomy. Similarly, another study has confirmed that age at least 70 years was an independent predictor of POT after aortic aneurysm repair surgery (18).

This study showed that aortic surgery was an independent risk factor to predict the occurrence of POT in our model. A significant relationship between aortic surgery and ventilatory dependency has been found in a previous study (13). Filsoufi et al. also observed that the highest incidence of respiratory failure was following aortic procedures and combined valve/CABG surgery (4). Of course, combined valve/CABG surgery was another surgical predictor of POT from our results. This factor has been demonstrated to be correlated to postoperative respiratory failure, and diabetes mellitus also has a correlation with respiratory failure (3). A study of the population eligible for CABG surgery indicated that patients with diabetes were more likely to have lower vital capacity parameters than those without diabetes (19). Moreover, patients with diabetes had a higher risk of having pulmonary complications, such as postoperative longer duration of ventilation and higher frequency of reintubation, and that the respiratory function was impaired in the course of the diabetic disease might explain it (20).

The presence of COPD in patients with cardiac surgery in our study was independently associated with the application of tracheostomy. COPD increased the risk of respiratory complications after cardiopulmonary bypass and was found to be related to extubation failure after cardiac surgery (21, 22). Preoperative disease processes, such as COPD, pulmonary hypertension, and severe left ventricular dysfunction, were surrogate factors for insufficient cardiopulmonary reserve, which increased the possibility of early postoperative extubation failure and prolonged mechanical ventilation (22). Pulmonary edema was also a factor causing respiratory insufficiency, and preoperative pulmonary edema was an independent predictor for POT in cardiac surgical patients. Hornik et al. showed cardiac surgery and cardiopulmonary bypass procedure could also cause inflammation and lung ischemia-reperfusion injury, leading to permeability pulmonary edema (23). Interestingly, a study of a virus-infected population found that pulmonary edema could independently predict the tracheostomy requirement (24).

The score model built based on the variables described above could assess the risk of POT for the cardiac surgical population. The endpoint of our study was treated with POT or no tracheostomy in patients who underwent cardiac surgery. However, many studies mainly focused on the timing of tracheostomy. The effect of early tracheostomy or prolonged intubation with possible late tracheostomy on the outcomes of mechanically ventilated patients has been a matter of debate. Trouillet et al. conducted a randomized trial and suggested that early tracheostomy provided no benefit in altering important clinical outcomes (25). While, Devarajan et al. indicated that early tracheostomy was associated with improved prognosis (26), and the differences in study results might be caused by different timing definitions of prolonged mechanical ventilation or tracheostomy. Overall, however, the outcomes of the cardiac surgical population with POT treatment were worse than those without POT. Therefore, our preoperative score prediction model was still meaningful for the management of cardiac surgical patients.

For the identified high-risk population at an early stage, early intervention may reduce the risk of requiring tracheostomy. Morimoto et al. suggested that prophylactic administration of sivelestat at the beginning of cardiopulmonary bypass could lead to better postoperative pulmonary function, thereby shortening the extubation time and decreasing the need for POT (27). A randomized controlled trial found that the cardiac surgical patients mechanically ventilated with a reduced tidal volume had reduced requirement for postoperative reintubation (28). In addition, the cardiopulmonary bypass has long been considered as a factor to produce diffuse lung injury, and the strategies, such as off-pump CABG use, heparin-coated circuits, and intraoperative ultrafiltration, could minimize this effect (29). However, for the low-risk patients, although there is also the possibility of failing to remove intubation early, our model may assist clinicians in making decisions, and perhaps these patients can avoid unnecessary additional invasive procedures, such as tracheostomy. Our scoring model may also provide meaningful information for the clinician to explain the illness condition to patients and their families.

Some limitations were however present. First, our study was a retrospective single-center design with a possible sample bias. Furthermore, due to different practices for tracheostomy support or families of patients differing in willingness to agree to additional surgery after cardiac surgery, our results and risk score model might not be generalizable to other centers. Second, our study results have not been externally validated, and the scoring model we constructed should be prospectively validated on cardiac surgical patient groups of other institutions. Third, although the tracheostomy was performed when estimated need for prolonged mechanical ventilation in our study population, there was no specific standard for tracheostomy. There might be some patients who should be treated with tracheostomy before death but did not actually undergo tracheostomy, and the subjective consideration to perform the tracheostomy or not was also an important limitation.

## Conclusion

In this study, the requirement of tracheostomy after cardiac surgery was indicative of a high-risk set with a significant postoperative burden. Our study identified significant predictors for POT in patients who underwent cardiac surgery and established a 9-point risk score to predict the need for POT. The model had good predictive performance and the three created risk intervals can be used for risk stratification, which may help surgeons to prevent the development into a severe state requiring POT in clinical practice.

## Data Availability Statement

The original contributions presented in the study are included in the article/Supplementary Material, further inquiries can be directed to the corresponding authors.

## Ethics Statement

The studies involving human participants were reviewed and approved by the Medical Ethics Committee of Tongji Medical College, Huazhong University of Science and Technology (IORG No. IORG0003571). Written informed consent for participation was not required for this study in accordance with the national legislation and the institutional requirements.

## Author Contributions

DW, YD, and SW participated in study design and contributed to the writing of the manuscript. DW, YS, SL, and HW contributed to data curation and analysis. XH, AZ, LW, and XD contributed to the revision of the manuscript. All authors read and approved the final manuscript.

## Funding

This study was supported by the National Natural Science Foundation of China (Grant Nos. 81800413, 82060092, and 81801586); Natural Science Foundation of Hubei Province (No. 2020CFB791), and Natural Science Foundation of Xinjiang Uygur Autonomous Region of China (No. 2020D01C181).

## Conflict of Interest

The authors declare that the research was conducted in the absence of any commercial or financial relationships that could be construed as a potential conflict of interest.

## Publisher's Note

All claims expressed in this article are solely those of the authors and do not necessarily represent those of their affiliated organizations, or those of the publisher, the editors and the reviewers. Any product that may be evaluated in this article, or claim that may be made by its manufacturer, is not guaranteed or endorsed by the publisher.

## Abbreviations

AUC: area under the receiver operating characteristic curve
CABG: coronary artery bypass grafting
COPD: chronic obstructive pulmonary disease
CI: confidence interval
ICU: intensive care unit
POT: postoperative tracheostomy
ROC: receiver operating characteristic.
