# Supplementary material for: A Predictive Scoring Model for Postoperative Tracheostomy in Patients Who Underwent Cardiac Surgery
Source: Front Cardiovasc Med. 2022 Jan 28;8:799605. doi: 10.3389/fcvm.2021.799605 (PMC8831542; doi:10.3389/fcvm.2021.799605)
Supplement: Supplementary file 1 [file Table_1.DOCX]

Supplementary table 1. Comparison of intraoperative variables in patients with and without POT after cardiac surgery

| Variables | All patients  n = 5,323 (%) | Without POT  n = 5,195 (%) | With POT  n = 128 (%) | *P* value |
| --- | --- | --- | --- | --- |
| Cardiopulmonary bypass time (minutes) | 105 (78, 142) | 105 (78, 140) | 163 (110, 231) | <0.001 |
| Aortic cross clamp time (minutes) | 69 (46, 95) | 68 (46, 94) | 100 (65, 134) | <0.001 |
| Intraoperative transfusion of red blood cells (units) | 1 (1, 3) | 1 (1, 3) | 7 (5, 8) | <0.001 |

Abbreviations: POT, postoperative tracheostomy.
